# A Model for Mission Dentistry in a Developing Country

**DOI:** 10.3389/fpubh.2017.00119

**Published:** 2017-08-02

**Authors:** Jan Hexamer Tepe, Lawrence J. Tepe

**Affiliations:** ^1^Dentists in Private Practice, Cincinnati, OH, United States

**Keywords:** oral care, dental mission work, school-based prevention, developing countries, volunteerism

## Abstract

Each year many dentists embark on mission trips to foreign countries. This article shares what one group learned in their journey over the course of 17 years to bring oral health to a rural community in Honduras. The group began by delivering acute dental care, but soon realized that this treatment would never change the status of oral health in the community. Year by year they learned what worked and what did not. A school-based dental prevention program was initiated using proven preventive techniques to demonstrate to the community that prevention of oral disease was possible. As of 2015, the school-based program had grown to over 10 schools and nearly 1,000 children had benefited from this program. Children in the program received all necessary treatments for the prevention and treatment of dental caries. As importantly, they and their families learned to understand how to be responsible for their own dental needs. In conclusion, it is possible to effect long-term change in a developing country by focusing on prevention of oral problems rather than focusing on the extraction hopeless teeth. The good intentions, time, and financial resources of volunteers can be put to best use by first learning about the needs and wants of a particular community. The authors recommend that volunteers partner with local health-care providers and research what other organizations are currently doing in their country of interest.

## Introduction

Each year, hundreds, if not thousands, of dentists participate in mission trips to foreign countries with the intent of “doing good” (1, 2). This article presents the evolution of a dental prevention program in Honduras, how the program achieved success over the course of 17 years, how this model can be applied in other third world countries, and how all involved in oral care in third world countries might collaborate their efforts.

## Background

Through the non-governmental organization (NGO) Shoulder to Shoulder (StS), dentists have delivered dental care in the remote village of Santa Lucia in Intibucá, Honduras since 1999. StS was founded in the early 1990s for the delivery of medical care (3). The medical personnel quickly realized that besides chronic medical ailments, many of the health problems resulted from the lack of oral care. Rampant caries and periodontal disease caused significant pain and suffering with time lost from both school and work. The first group of dentists was able to provide extractions under crude conditions that lacked proper sterilization facilities. In the years that followed, the physical conditions for dentistry improved with the installation of typical equipment such as air compressors, central suction, and autoclaves. In addition to prophylaxes and extractions, dentists were able to provide amalgam and composite restorations as well as anterior endodontia.

Like many mission groups, the StS model depended upon the labor of volunteer dentists from the US. Quickly, we realized that the need and demand for dental care far exceeded our resources, especially when our care was limited to several weeks of care each year. It was wasteful for our facilities to sit idle when we were not in the country. Without any local dentists, we trained a young lady who provided dental education and simple rubber cup prophylaxes to children. We also taught the resident Honduran physician how to extract hopeless teeth. Still, it was discouraging to see the ever increasing number of children with early childhood caries, teenagers with rampant caries, and adults missing many teeth. We investigated how we might make fluoride available to the population and considered vehicles such as fluoridated water, topical applications, and fluoridated salt and milk (4–8). After searching the literature for an efficacious, safe, and controlled delivery system for fluoride, we began a program centered on education, daily brushing, and three varnish treatments yearly (9, 10). We wanted to prove to both ourselves and the community that dental decay could be controlled and that tooth pain and loss were neither expected nor inevitable.

As we had no caries baselines in the communities of Concepción or Santa Lucía in the Department of Intibucá prior to making dental care available, we selected a similar community (the public school in Los Pinares in the Department of Intibucá) where no dental care was available. It should be noted that even though a dentist was available in Concepción and Santa Lucía, few if any of the children in the schools in our proposed study area had ever seen a dentist. School children in Los Pinares were examined by one dentist (Jan Hexamer Tepe) using a modified version of the World Health Organization (WHO) Health Assessment Form (11). Children aged 5, 6, 12, and 15 years were examined. From this small pilot study (*n* = 134), we found that the DMFT number for 12-year olds in this community was 3.7, the same as the WHO number of 3.7 for Honduras (12). Children 5–6 years old had a DMFT of 8.7. The teeth most affected by caries in our study were mandibular molars, maxillary molars, and maxillary central incisors, respectively. By age 5–6, 95% of the children demonstrated dental caries. At the age of 12 and 15, 82% of children demonstrated dental caries, none of which had been treated (Tepe JH. *2008 Caries Study – Honduras. Unpublished Report of Pilot Study*).

One of the greatest difficulties in bringing oral treatment and preventive care to an uneducated population is the lack of understanding that oral disease is preventable. In rural Honduras, it is normal to see people of all ages with visibly large carious lesions in their anterior teeth as well as missing teeth. Dental care is often limited to the extraction of painful or hopeless teeth, and this treatment is often not provided by a dentist. Professionally trained dentists can be found in public clinics in larger towns and private offices, but they lack typical amenities which would be expected in the US. During 1999 to 2014, we often visited dentists in both public and private settings. We observed in the early 2000s that it was rare to find working X-ray machines for either type of office. Units, chairs, and lights were old and cobbled together. “Sterilization” was achieved by cold sterilizing solutions or by boiling. In one public clinic, the dentist was mixing amalgam by mortar and pestle and the dental light had been retrofitted to hold a fluorescent screw in bulb. In areas where there is no public health or private practice dentist, there are non-professionally trained “lay-dentists” (13). These individuals usually limit their services to extractions and the fabrication of acrylic partials and dentures and often work out of their homes. Both the Honduran government and organized dentistry in Honduras, El Colegio de Cirujanos Dentistas de Honduras (The Colegio), are attempting to curb these practices, but they still exist in remote areas where access to a professional is lacking.

Honduras, like many other third world countries, is changing quickly. In the towns where our clinics are located, there was no electricity until the mid 1990s. Travel by horses, donkeys, carts, and on foot was common. The chores of daily living such as the planting and harvesting corn on steep mountainsides, preparation of food, hauling water, care of animals and children, sewing, and washing clothes by hand consumed the day. There was little knowledge of the outside world and many people had never traveled farther than the next town. Government schooling was and continues to be provided for children only to the sixth grade, but frequently the children were removed from school as soon as they became large enough and strong enough to help with chores at home and in the fields. In the mid 1990s when electricity came to the frontier, everything changed. By then, many families had a relative working in the US sending money back home to Honduras. Money from the US gave people the purchasing power to buy televisions and pickup trucks. The countryside became littered with the trash of progress-plastic bags and bottles, foil snack bags, and cellophane candy wrappers. Diets changed as penny candy, soda pop, and snack foods became affordable and available (14–16). But still, children suffered from malnutrition, diarrhea, parasitic worms, and dental decay. Progress brought them so many “things” but not the education to know how to use and manage their new-found materialism.

Today, it is rare to find the donkey carts and horses of 20 years ago. Pickup trucks and small open air three-wheeled taxis now transport people. Town markets are filled with clothing and household goods imported from China. Cell phones are everywhere. The country has developed in some aspects, but oral care and prevention are still lacking. People now watch TV and see healthy teeth. They see their poverty as they never did before and they also want better lives and opportunities for their children.

## The Current State of Dentistry

Today, there are four dental schools in Honduras. The public university is the Universidad Nacional Autónoma de Honduras, located in Tegucigalpa with a smaller branch in the city of San Pedro Sula and two smaller Catholic dental schools also located in Tegucigalpa and San Pedro Sula. The government schools are tuition free and open to students who have finished the equivalent of high school and have passed a written exam. The two government-run schools together have an enrollment of 2,300 students, with approximately 330 dentists graduating each year. Dental school is 6 years with a seventh year of required community dental service. Once a student has graduated, their opportunities for employment are to work in private practice, a government health clinic, for an NGO, or for a private company. The Minister of Health has not had a position open for a new graduate for the past 2 years and there is currently a surplus of unemployed dentists. So, while there is great need for dental care, the country lacks employment opportunities for these new graduates. When we questioned why a young person would wish to spend 7 years of their life learning a difficult profession when employment opportunities are so dismal, we learned that students desire the respect and status afforded to the profession of dentistry.

Shoulder to Shoulder hired our first Honduran dentist in 2003, and in doing so, we learned that the laws regarding the practice of dentistry for both Hondurans and foreign volunteer dentists are complex and not easily available (17). For example, the Minister of Health and The Colegio require that visiting dentists register with them prior to working in Honduras by supplying a copy of the dentist’s diploma and a copy of the dentist’s current registration. Then, a visiting dentist is mandated to work under the supervision of a licensed Honduran dentist. Honduran dentists who are practicing outside of their own private practice are limited to working only 6 h per day. Prophies, extractions, and restorations of amalgam and composite are the services typically available. Endodontia, crowns, and fixed prosthesis are not available at public clinics. Toothpaste is widely available in stores, markets, and even sold by vendors in the aisles of local buses. Fluoride varnish, however, is rarely used and toothpastes with higher levels of fluoride such as Prevident are not available. Systemic fluoride *via* water, salt, or milk is not currently available (18, 19). Education about dental care is done primarily through the marketing efforts of Colgate-Palmolive who supplies some schools with posters, pamphlets, coloring books, brushes, and paste.

## Spreading the Word of Preventive Care

With these thoughts in mind, we set out to prove to both ourselves and to the people in the communities served by StS that oral health was possible. The notion of visiting a dentist for a checkup was a completely foreign idea. People sought care only when something hurt. Even when people could see holes in their teeth and other obvious oral problems, they did not seek treatment. This became a problem since our mission was to bring preventive care and avoid loss of teeth. In response, we reached out to people through radio announcements that included topics such as the care of babies’ and children’s teeth, the role of diet on oral health, the importance of home care, and how a dentist can help prevent tooth loss. Our Honduran dentist also gave talks about oral care at churches, schools, civic meetings, and fairs. Still, people refrained from seeking preventive care.

## Proving That Prevention is Possible

In 2010, we decided to demonstrate the effectiveness of preventive care in a controlled environment by initiating a school-based program. From the pilot study, we knew that by the time a child is 6 years old, the majority of deciduous teeth have already been affected by dental caries. We sought to preserve the permanent teeth as soon as they erupted by giving each child three fluoride varnish treatments yearly, sealing all permanent molars, and restoring any carious permanent teeth. We did not attempt to restore carious deciduous teeth unless they were abscessed or painful. We sought a school where children were the neediest and where the school director, teachers, and parents were supportive of the program. The parents and teachers were required to attend a session with the Honduran dentist who explained the program in detail. The adults were given education about how to care for teeth, the importance of diet, and their responsibilities. Teachers were taught how to apply the varnish and were supplied with gloves, gauze, and varnish. Children were excused from school three to four at a time and transported to the dental clinic for as much treatment as was feasible in 1 day. Children brushed daily at school under the supervision of their teachers and were given a second brush to use at home. The dentist and the assistant visited each classroom on a regular basis to reenforce daily habits.

We started with one school in the area of Concepción in the Department of Intibucá. As soon as all of the children in this school had received comprehensive treatment, a second school was added. As of 2015, the program included a total of 11 schools in the communities of Concepción and Santa Lucía (both in the Department of Intibucá) with 974 students. Meticulous records have been kept, which indicate how many sealants, restorations, extractions, prophylaxes, and varnish treatments have been delivered. In January, 2014, two of the original dentists involved with the inception of the program visited all of the schools in the program. We observed children with healthy mouths and no evidence of dental caries in permanent teeth. By comparison, 95% of children aged 5 and 6 and 82% of children aged 12 through 15 had visually detectable caries in the pilot study in a similar community. This program has been so successful that some children moved to live with relatives who reside within the boundaries of a school that has the prevention program, solely for the dental care. Now, other schools, parents, and communities are eager to have similar programs for their children.

## How to Expand This Model

The challenge for an NGO such as StS is finding the resources and materials to expand this type of prevention program. We know how to succeed and we have demonstrated to several communities that oral health is achievable. Our work has been done through the dedication of two Honduran dentists who have delivered the majority of treatment to the children. Financial support comes from a small circle of friends, relatives, patients, and dental colleagues of the original dentists who began the dental clinic with StS in 1999. Our organization has no corporate sponsorship and no major donor to sustain our operations. We have been fortunate to be able to purchase some of our supplies through US dental companies and charitable organizations that sell surplus materials at cost. However, airline restrictions now severely limit the transport of materials that are needed to sustain preventive programs such as ours. Many supplies are available for purchase in Honduras, but prices and quality are often not comparable with what is obtainable in the US. The cost of the preventive school program is about $50 US per child per year, which does not include the cost of transportation of children to the clinic or the startup costs to establish a clinic with necessary dental equipment.

In October, 2014, Dr. Jan Tepe visited dental leaders in Honduras to share the success of this program and to learn what other efforts are underway to improve oral health in Honduras. Among the leaders were Dr. Carlos Aguilar, director for dental care in Honduras who reports directly to the Honduran Minister of Health, Dr. Lourdes Murcia, Dean of Facultad de Odontologia of the Universidad Nacional Autónoma de Honduras in Tegucigalpa, Dr. Ernesto Jimenez, of the Departamento de Vinculación of the Nacionál Autonoma de Honduras, board members of El Colegio de Cirujanos Dentistas de Honduras, and Dr. Juan Burgos, Director of Professional Relations for Colgate-Palmolive Honduras. In each of these meetings, we explored what is currently being done to improve oral care in Honduras. We learned that students in education, medicine, and nursing learn nothing about oral care or the prevention of oral diseases. We learned that incorporating fluoride into salt as proposed by the Pan American Health Organization (PAHO) (19, 20) has not been implemented due to cost. Searching the literature, we found school programs described in minute detail by WHO for the promotion of oral health that have never been successfully implemented in Honduras (21). Most significantly, we learned that to the knowledge of each individual and group with whom we met, none was aware of any other successful long-term school-based prevention program such as ours. WHO, PAHO, and governmental agencies repeatedly hold conferences and write papers with goals, proposals, and plans for the reduction of oral disease in third world countries (16, 22–27). The implementation of proposed plans seldom happens. Too often, the will to succeed is thwarted by lack of staff resources and funding (28). Oral disease is a complex disease that involves educating people, changing their attitudes and behaviors, and instilling individual acceptance for responsibility. It involves parents, children, teachers, physicians, nurses, and even public officials to advocate for limiting the availability of cheap and unhealthy food and drink and to make sources of systemic fluoride available.

What are possible solutions to the lack of oral health in third world countries? We have shown that a modest investment in prevention can yield dramatic improvements in oral care. Systemic fluoride has been hailed as one of the 10 greatest advances in oral care in the twentieth century (29) yet in Honduras, a country of young people with a median age of 21.57 (30); where more than 38% of the population is under the age of 15 (31) there is no widespread source for systemic fluoride. Prevention is the most cost-effective means for combating oral disease and is best started with education. Education does not require special equipment and is the essential key to understanding and changing behavior. Mouths can be restored, but until we teach patients how to care for their own mouths, understand the cause of their problems, and then be responsible for themselves, they will return with the same problems. Next, professional staff is needed to diagnose and treat oral problems. Hundreds, if not thousands of dentists embark on mission trips each year (32), yet few are providing education along with the restoration of teeth. Extracting teeth simply reinforces the notion that one goes to the dentist for extractions. How much better it would be for dental volunteers to partner with a public health dentist and supply them with restorative materials. Mission groups can also help by employing young Honduran dentists. Where there is no clinic available, portable equipment could be shared by multiple mission organizations and stored in-country and thereby avoid the problems and expense of transporting items back and forth. Volunteer dentists could go into the schools to start the same type of program that StS has initiated. To facilitate a program of partnerships, we also propose the development of a clearing house to connect dentists and mission organizations to one other and also to individual communities. A website through the American Dental Association, the WHO, or PAHO might be logical sites for collaboration. In this way volunteers could learn beforehand what the needs are, what desire for treatment exists, what legal requirements exist, and what dental facilities and supplies are available (33). Organizations and individuals engage in mission trips with good intentions, but without coordination, valuable time and resources are wasted. Collaboration, with commitment to a long-term presence, can provide the opportunity for real change. Other short-term efforts often bring a sense of goodwill to the well-meaning volunteer, but do little to improve oral care or effect change (34).

## Conclusion

It is possible to achieve oral health among children in a third world country through intensive education of children and their parents with concomitant restorative dentistry. For oral care providers who are affiliated with mission groups we suggest that they: learn about the laws of the country, talk to native dentists to determine their needs and challenges, and aim for sustainable results through education and prevention. In countries where dental professionals are underemployed, we recommend using resources to employ native professionals and supply them with materials and equipment. Countries wishing to receive help from non-profit organizations could facilitate relief efforts by sharing information about the current health status of their population and making information about current or proposed projects available publicly.

We have been able to achieve success with the limited resources of a small non-profit organization. How much more could be achieved if ALL resources for the indigent could be shared and coordinated? The parties involved in improving oral health in third world countries (hereafter called the “host” country) include university outreach programs, non-profit mission organizations, host country dental organizations, government sponsored organizations such as the WHO and PAHO, as well as programs initiated and funded by the host countries themselves. These groups invest millions of dollars each year to support their individual projects, but there is little sharing of what each group is actually doing. Efforts to learn what is available and the activities of each organization are difficult if not impossible to find *via* the internet or even in face to face meetings with officials. This fragmentation of knowledge and lack of coordination can result in the duplication of services and wastes valuable resources. In our efforts to establish a prevention program we encountered many obstacles as a result of this lack of transparency. The ADA is to be commended for their encouragement of mission work and the information they give prospective volunteer dentists through their website. We urge other parties such as NGO’s, universities, WHO, PAHO, and Ministers of Health who are involved in the planning and execution of oral care programs to make their work publicly available so the greatest number of people can receive the best care possible.

Mission work can be exceedingly rewarding for volunteers. It is even more rewarding if it brings lasting change to patients and their families. We encourage dental professionals to be involved in care for the indigent, especially in places in the world where no care exists. We challenge organizations to share their work for the benefit of all who work for the goal of improving oral care.

## Author Note

JT is an Assistant Professor in the Division of General Practice and Materials Science, The Ohio State University College of Dentistry, Columbus, Ohio, USA.

## Author Contributions

JT has been part of the dental team working in Honduras for 17 years and provided treatment, determined rationale, researched best practices and modes for delivering care, as well as helped to install equipment, maintain inventory, and procure funding for the project and supplies. LT was also directly involved with dental care for 17 years, acquired equipment for the dental clinics, installed equipment, and oversaw the hiring and supervision of the Honduran dentists and staff.

## Conflict of Interest Statement

The authors declare that this work was conducted in the absence of any commercial or financial relationships that could be construed as a potential conflict of interest.
